# How Toxic Workplace Environment Effects the Employee Engagement: The Mediating Role of Organizational Support and Employee Wellbeing

**DOI:** 10.3390/ijerph18052294

**Published:** 2021-02-26

**Authors:** Samma Faiz Rasool, Mansi Wang, Minze Tang, Amir Saeed, Javed Iqbal

**Affiliations:** 1 Postdoctoral Station of Statistical, Guangzhou University, Guangzhou 510006, China; samma@i.shu.edu.cn; 2 Entrepreneurship Institute, School of Innovation and Entrepreneurship, Guangzhou University, Guangzhou 510006, China; 3 School of Management, Guangzhou University, Guangzhou 510006, China; 4 Institute of Administrative Sciences, University of the Punjab, Lahore 54000, Pakistan; aamir.ias@pu.edu.pk; 5 School of Education, Guangzhou University, Guangzhou 510006, China; javed@e.gzhu.edu.cn

**Keywords:** toxic workplace environment, organizational support and employee well-being, small and medium-size enterprises

## Abstract

This study explores the effects of a toxic workplace environment (TWE) on employee engagement (EE). Building on conservation of resources (COR) theory and organizational support theory (OST), this study proposed a research model. In this research model, a toxic workplace environment negatively affected employee engagement, directly and indirectly, through organizational support (OS) and employee well-being (EW). In this study, we used a quantitative research approach, and data were collected from 301 workers employed in the small and medium-size enterprises of China. To estimate the proposed relationships of the research model, we used partial least squares structural equation modeling (PLS-SEM 3.2.2). The results of this study confirmed that a toxic workplace environment has a negative impact on employee engagement. Moreover, the findings of this research confirm that organizational support and employee well-being significantly mediate a toxic workplace environment and employee engagement. The conclusions of this study are as follows: First, the direct relationship between a toxic workplace environment and employee engagement confirms that if employees are working in a toxic environment, they will spread negative feelings among other co-workers. The feelings that come with a toxic workplace environment, i.e., harassment, bullying, and ostracism, can be detrimental and lead to unnecessary stress, burnout, depression, and anxiety among the workers. Second, employee well-being will affect employee behaviors that enhance employee engagement with the work as well as with the organization. Third, organizational support also increases employee engagement with the work as well as with the organization. So, it is also confirmed that when workers perceive the support from the organization, their sense of belonging to the organization is strengthened.

## 1. Introduction

Over the past three decades, China has witnessed tremendous industrialization, urbanization, and economic growth. This growth has partly stemmed from the rapid development of small and medium-size enterprises (SMEs) within China. Currently, small and medium-size enterprises employ 80% of the workforce in China [1,2]. One reason why SMEs have flourished this way is that small and medium-size enterprises have fewer regulations imposed on them as compared to large enterprises. However, employees belonging to SMEs have to suffer from a lower pay and a high level of toxic workplace environment, such as workplace harassment, workplace bullying, and workplace ostracism [3]. Such an environment is a significant detriment toward employee motivation and engagement, and prior studies have shown that a toxic workplace environment in small and medium-size enterprises plays a negative role toward employee engagement [2,4].

Employee engagement, which refers to a commitment demonstrated by employees toward their job and organization, has become an important asset for small and medium-size enterprises that seek to adapt to an uncertain environment [5]. Therefore, there is increased attention among organizational behavior theorists toward the personal and situational factors that influence employee engagement [6]. Such research has increasingly focused on personal factors, in a social or a group context, that tend to influence engagement levels [7]. Other variables, such as organizational culture [8], relationship with bosses, and job features [9], are also routinely researched.

In this regard, the overall organizational environment is a matter of great concern. A toxic workplace environment refers to the cruel and often violent treatment of persons, and it jeopardizes employee safety and health [10]. The impact of a toxic workplace environment is perhaps felt within every organization, but due to personal reasons, very few of the workers are willing to lodge formal complaints against such behavior [11]. This avoidance and silence by victims of a toxic workplace environment make such incidents difficult to be noted and studied by researchers [12]. However, it is unanimously acknowledged that victims of violence suffer from a lack of well-being. Employee well-being here refers to a feeling of happiness felt by people based on a sense of security and satisfaction [13]. According to Maslow’s theory of needs, security is the main concern for people, and insecurity is not applicable to other higher-level needs [14]. A toxic workplace environment, however, is a climate factor that demolishes a person’s sense of security and, thus, is bound to have a negative impact on well-being. In addition, organizational support is an important source of employee engagement. Although a lot of studies have investigated the psychological processes that promote employee engagement [15,16,17], there has not been a clear distinction of organizational characteristics that contribute to cognitive processes that are supportive of innovation and individual development [15,18]. To explore these factors of employee engagement based on this research gap, this study proposes an empirical model that tests the negative effect of a toxic workplace environment (i.e., harassment, bullying, and ostracism) on employees through individual emotional processes, which include employee well-being and organizational support.

This proposed model advances several theoretical perspectives. First, by assessing the relationship between a toxic workplace environment and employee engagement, considering negative effects, this research studies the factors of a toxic workplace environment that are not found in small and medium-size enterprises operating in the vicinity of China. Such kind of research has not been conducted in previous studies. Previous studies have only focused on positive environmental factors and have ignored negative environmental factors. Second, the use of conservation of resources (COR) theory to understand employee engagement is used for the first time in the literature. COR theory covers two basic principles involving the protection of resources from being lost. The first principle is called the primacy of resource loss. This principle states that it is more harmful to individuals to lose resources compared to when there is a gain of resources. What this means is that a loss of pay will be more harmful than the same gain in pay would have been helpful. The second principle is known as resource investment. This principle of COR states that employees tend to invest in resources in order to protect against resource loss, to recover from losses, and to gain resources. So, when employees’ resource bases become depleted through their exposure to adverse work situations, such as harassment, bullying, and ostracism, they may avoid positive behaviors, which negatively affects employee engagement [19]. Similarly, according to the second principle of COR theory, employees invest in resources to prevent future resource losses, which positively enhances employee engagement [19]. The study also eliminates employee engagement on the basis of organizational support theory, which pays significant attention to the psychological process of employees [20]. Finally, this study examines the mediating effects of employee well-being and organizational support on the relationship between a toxic workplace environment and employee engagement, and the findings of the study suggest that employee engagement is not an absolute utilitarian behavior and also arises out of an unconscious organizational citizenship activity. Hence, on the basis of the above discussion, we generate the below-mentioned three research questions (RQs):

**RQ1:**  How does a toxic workplace environment influence employee engagement?
**RQ2:**  How does organizational support intervene between a toxic workplace environment and employee engagement?
**RQ3:**  How does employee well-being intervene between a toxic workplace environment and employee engagement?

This paper is organized as follows. In Section 2, we present a literature review. Section 3 presents hypotheses development, while Section 4 presents the research methods, and Section 5 explains the statistical analysis of this study. Section 6 presents the discussion, and Section 7 explains the concluding remarks. Finally, the last part of this study presents the limitations and future research directions.

## 2. Literature Review

### 2.1. Toxic Workplace Environment

A toxic workplace environment is a description of the relationship between workers and the workplace [21]. There are two types of workplace environments previously identified by researchers: a collaborative work environment and a toxic work environment. A collaborative work environment is a friendly place with the right mix of pleasure, involvement, and organizational citizenship behavior [22]. A toxic workplace environment is defined by narcissistic behavior; offensive, and aggressive leadership; threatening behavior from managers and co-workers; and harassment, bullying, and ostracism. A physical and mental imbalance is regularly observed in a toxic workplace environment, which is alarming due to the deep-rooted grounds for high levels of stress and burnout and is a source of psychological strain on the employees’ health. Work pressures generate counter-productive work behavior at the workplace and ruin the efficiency of the organization [23]. After an extensive literature review and based on COR theory, this study focuses on the following factors of a toxic workplace environment: (i) workplace harassment, (ii) workplace bullying, and (iii) workplace ostracism. These factors are demarcated as follows: (i) workplace harassment refers to peers and supervisors threatening and poorly mishandling the workers [24]; (ii) workplace bullying means an individual is mistreated by a group or an individual in any situation, such as cyberbullying or harming the peers and stakeholders at work [25]; and (iii) workplace ostracism is defined as workplace loneliness of the workers due to their peers, family, stakeholders, and bosses [26]. The outcomes of workplace ostracism increase employee turnover and job dissatisfaction [2]. Moreover, previous researchers and COR theory also suggest these three factors reported above create toxic environments in organizations that reduce work performance and employee engagement [27].

### 2.2. Employee Engagement

Employee engagement is a source of a physical and emotional connection between employees and the organization [28]. It aligns employees’ personal goals with the vision of the organization, which increases the productivity of the employees and, hence, the organization [29]. An engaged employee is well balanced and emotionally connected with the vision and mission of the organization, which portrays and governs the involvement of the employee in the organizational objectives [30]. An engaged employee will work with a progressive attitude, which will build the repute and value of the organization. Organizations cultivate environments to encourage and indulge high engagement of employees, and engaged employees are enthusiastic for all support from their organizations [31]. Organizations define well-equipped designs to engage employees, which aligns worker goals with those of the organizations. Employee engagement is a positive method to avoid burnout and disengagement of the employees and indulge their emotions into positivity and patronized ethical behavior at the place of work.

### 2.3. Organizational Support

Organizational support refers to the course of perception and beliefs on behalf of the employee, where it is believed that the organization has a deep concern for employee well-being [32]. Organizational support facilitates instrumental, social, and emotional support [33]. Organizational support has been examined alongside various other variables, all of which support the view that organizational support reduces worker stress and burnout [34,35]. Accordingly, informal support is more helpful, when provided, as compared to formal support from an official senior [35].

### 2.4. Employee Well-Being

Apprehension, illness, depression, and fatigue are some of the aspects of a lack of mental health and the overall well-being of any human being. Likewise, headaches and muscular aches are signals of physical ill-health. An employee’s well-being is an accelerator for organizational success, saves the organization from lower productivity, and decreases poor health insurance costs. Progressive organizations have to make sure that their programs have health outcomes for the overall well-being of their employees. The physical environment of the workplace and organizational climate are some of the important aspects of employee well-being. An organization communicates its agenda for employee well-being, as it is obliged to do so under corporate social responsibility initiatives [36]. The results of previous studies lead to the hypothesized relationship between a better quality of employee well-being, optimistic behaviors, and intentions [37,38,39]. So, it is proposed that corporate social responsibility initiatives help to create a positive work environment that promotes employee well-being in return and prompts active participation for green behavior.

## 3. Hypotheses Development

### 3.1. Toxic Workplace Environment and Employee Engagement

There is a lot of evidence from prior studies that show a significant relationship between a toxic workplace environment and employee engagement [28,31]. According to Bakker and Albrecht [29], an engaged employee is a motivated, self-guided, and contributive member who represents a valuable addition to the human capital and promotes organizational growth and development. Das and Mishra [40] categorize employee engagement into two types: job engagement and organizational engagement. Job engagement leads to employee commitment, which directly deals with dedication and work performance, which routes to organizational development. Organizational engagement is interlinked with employee commitment and employee loyalty. The prior literature supports the view that the impact of at oxic workplace environment on the involvement of the individual, job satisfaction, and enthusiastic characteristics for work is negative, while employee engagement and organizational engagement are adversely affected [41]. Moreover, COR theory also supports the negative relationship between a toxic workplace environment and employee engagement. So, as a result, the construct of employee engagement is significantly associated with a toxic workplace environment and can be hypothesized as follows:

**H1.**  *A toxic workplace environment is negatively related to employee engagement.*

### 3.2. Mediating Effect of Organizational Support

The presence of a toxic workplace environment is found to have negative effects on employee outcomes, such as stress and engagement. However, certain mediating variables can mitigate these negative effects, and one such variable is organizational support. In a study on the toxic work environment and its relationship with work stress, Wang, Zaman [32] found that organizational support has a positive impact on employee output, which improves employees’ commitment and performance at the workplace. It has also been established that when organizational support is provided to employees, their cognitive and emotional evaluation of their organization is strengthened [2]. In this view of a dyadic interaction between employees and their organization, it can be presumed that high levels of organizational support would allow employees to experience higher engagement levels, even if engagement levels are diminished by the presence of unfavorable work environment characteristics. Moreover, COR theory also supports the negative relationship between a toxic workplace environment and organizational support. Hence, on the basis of the above discussion, we proposed the below-mentioned hypothesis.

**H2a.**  *A toxic workplace environment is negatively related to organizational support.*

Organizational support has been proven to have a positive effect on employee engagement in several studies. Organizational support reflects an organization’s overall expectations of its members and recognizes the personal value of each employee [42]. The use of social exchange theory and organizational support theory has generally been used to explain the relationship between organizational support and employee engagement. Social exchange theory states that relationships evolve over time into trusting, loyal, and mutual commitments as long as both parties abide by the rules of the exchange. For example, when employees receive economic and socioeconomic resources from their organization, they feel obliged to respond in kind and repay the organization, thus helping to promote employee engagement [43]. Similarly, in the view of organizational support theory, when employees perceive support and care from their organization, they make active attitudinal and behavioral changes in order to achieve organizational goals. In other words, when employees feel that their organization is concerned about them, they would respond by becoming more engaged [44]. Thus, according to the above-discussed literature, organizational support is positively related to employee engagement.

**H2b.**  *Organizational support is positively related to employee engagement.*

**Figure 1:**
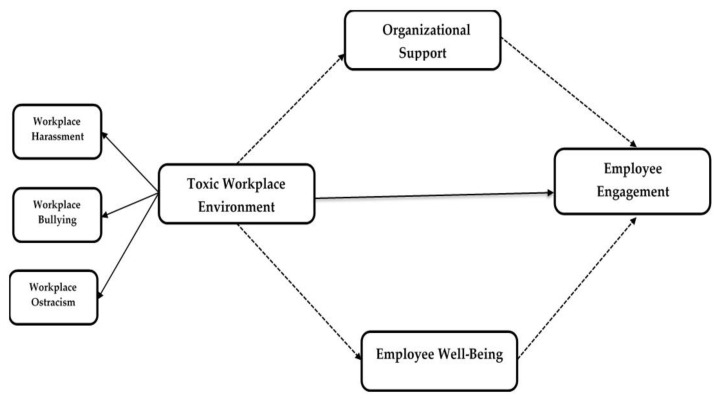
Research model. Dashed arrows indicate indirect relationships, and solid arrows indicate direct relationships.

Previous studies indicate that organizational support has a significant impact on workers’ output, their job commitment to the organization, and high productive work, transforming the organization and the effectiveness of the fundamental values of the organization [45,46,47]. Organizational support in the form of leadership support at the workplace has a positive impact on the dynamic behavior of the workplace [48]. Motivation in employees from organizational support leads toward more productivity [49]. According to organizational support theory, organizational support plays a significant role in employee engagement. For instance, the demand control support (DCS) model shows that mental health problems at work arise out of excessive pressures, low control, and low support [50]. This model shows the negative consequence of a toxic environment, but if supervisors and peers provide support to the workers, it will enhance employee engagement [30,51]. Our study also proposes a theoretical framework (Figure 1) based on organizational support theory and COR theory. This theoretical framework also indicates that organizational support can create a better workplace environment and can mediate between a toxic workplace environment and employee engagement [52,53,54]. Thus, on the basis of the above discussion, we proposed the below-mentioned hypothesis.

**H2c.**  *Organizational support mediates the relationship between a toxic workplace environment and employee engagement.*

### 3.3. Mediating Effect of Employee Well-Being

According to the prediction of a number of specific studies, a toxic workplace environment negatively influences employee well-being [55]. Previous studies have shown that the presence of a toxic workplace environment threatens the well-being of employees. For instance, numerous studies have suggested a positive association between workplace bullying and poor well-being. Rajalingam [56] observed that workplace bullying results in greater stress and lower satisfaction levels among employees who have been subjected to it. Thus, being bullied at work is a threat to psychological well-being as well, and employees who are subjected to bullying report greater levels of general and mental stress [57]. Similarly, workplace ostracism has also been negatively linked to employee well-being because it leads to enhanced levels of job tension and emotional exhaustion [58]. The above relationship is shown in the following hypothesis.

**H3a.**  *A toxic workplace environment is negatively related to employee well-being*.

Prior research indicates that high levels of employee physical and psychological well-being play a significant role in delivering some important organizational outcomes that are associated with high-performing organizations, such as employee engagement. The proposition that employee well-being is important in developing sustainable levels of employee engagement appears to have sufficient theoretical expectation and empirical research evidence [59]. Furthermore, He, Morrison [60] found that employees who reported higher levels of engagement were likely to benefit from a broadened allocation of psychological resources, one of which is employee well-being. Thus, the positive relationship of employee well-being with employee engagement is depicted in the following hypothesis.

**H3b.**  *Employee well-being is positively related to employee engagement.*

Moreover, prior researchers have also found that employee well-being motivates all employees, i.e., top-level, middle-level, and administrative staff [61]. According to the results of multiple studies, if an employee is more committed to his/her organization, it is obvious that said organization participates in employee well-being [62]. Moreover, it is also concluded that the quality of work life is deep rooted in the engagement of an employee toward the organization’s citizenship behavior [63]. Fotiadis, Abdulrahman [64] demonstrated that an employee would perform well if he/she has good concerns. So, if organizations care about their employees, employees will, in return, positively engage with the organizations. The below-mentioned hypothesis was proposed with this understanding. In addition, Figure 1 presents a comprehensive research model of this research. In this spirit, we also hypothesized the following.

**H3c.**  *Employee well-being mediates the relationship between a toxic workplace environment and employee engagement.*

## 4. Research Methods

In this study, we used a quantitative research approach. The online survey method was used for data collection. The reasons for online data collection were, first, it is a low-cost method to collect data [65]. Second, the response rate is usually higher than manual distribution of a questionnaire. Third, most of the data collection was done during the COVID-19 pandemic, during which the majority of employees were observing the lockdown period or were working from home. Hence, an online survey was the most appropriate strategy to collect responses. The study was cross-sectional in nature and was conducted from April 2020 to August 2020 in China based on a convenience sample approach. Hennessy and Patterson [66] suggested in their study that for survey analysis, authors first design the research instrument [67]. So, in this study, first, we developed a research instrument to collect the data.

### 4.1. Instrument Development

In this research, first, we designed a questionnaire for data collection, and the base was provided by the constructed hypotheses [66]. The questionnaire comprised 21 items scored with a 7-point Likert scale (1 = strongly disagree and 7 = strongly agree). A pilot study checked the instrument’s reliability and validity: 30 drafted questionnaires were distributed to the stakeholders, which included 10 academic professors, 10 PhD students, and 10 professionals, all of whom had sufficient knowledge of the research objectives. After a little fieldwork for the pilot study, some recommended changes were made to modify the instrument to meet the objectives. This action was considered a necessity before collecting information from the targeted population.

### 4.2. Data Collection and Sampling

Data were collected from workers working in small and medium-size enterprises within the Guangdong Province of China. A cover letter explained the purpose of the instrument, assured the respondents of the confidentiality of their responses, and informed them that the information collected would only be utilized for research purposes. Before data collection, we tested the reliability and validity of the questionnaire through a pilot study. Furthermore, through WeChat (a social network application) and emails, we distributed 500 questionnaires among senior managers, middle-level managers, and administrative staff and received a total of 324 filled responses. After further refinement, the completed sample size was 301.

### 4.3. Variables and Measures

This paper included three dimensions of a toxic workplace environment: workplace harassment, bullying, and ostracism. The independent variable toxic workplace environment consisted of eight items, and these items were adapted from Anjum, Ming [68] and Rasool, Maqbool [67]. The score was measured on a 7-point Likert scale (1 = strongly disagree and 7 = strongly agree). The detailed information about all items of the questionnaire is presenting in Appendix A. Cronbach’s alpha of the toxic workplace environment was 0.935. The items used in the study were considered valid because of their alpha value above the standard value of 0.70 and higher. So, the items we used in this research instrument are valid.

The scale for measuring organizational support was adapted from Wang, Zaman [32]. The scale included seven items and measured the responses on a 7-point Likert scale (1 = strongly disagree and 7 = strongly agree). The detailed information about all items of the questionnaire are presenting in Appendix A. Cronbach’s alpha of organizational support was 0.784. The standard value of alpha is 0.70 and higher. So, the items we used in this research instrument are valid.

The scale for employee well-being was adapted from Ahmed, Zehou [69], which also measured the responses on a 7-point Likert scale (1 = strongly disagree and 7 = strongly agree). The detailed information about all items of the questionnaire is presenting in Appendix A. Cronbach’s alpha of employee well-being was 0.843. The items used in the study were considered valid because of their Cronbach’s alpha value above the standard 0.70.

The items of employee engagement were adapted from Saleem, Shenbei [70], and all items of this variable were measured on a 7-point Likert scale (1 = strongly disagree and 7 = strongly agree). The detailed information about all items of the questionnaire is presenting in Appendix A. Cronbach’s alpha of employee engagement was 0.759. The standard value of Cronbach’s alpha is 0.70 and higher. So, the items we used in this research instrument are valid.

### 4.4. Respondents’ Summary

The questionnaire was distributed to 500 employees working for SMEs located in Guangdong Province, China. A total of 324 responses were received in return, of which 301 were used for this paper. In this study, we used descriptive statistics. The majority of the respondents in this research were male, i.e., around 54.44% males and 45.51% females. The proportion of respondents with a work experience above 15 years was 26.57%, with more than 10–15 years was 33.88%, and 5–10 years was 39.53%. Similarly, the positions from whom we collected data were senior managers (26.91%), middle-level managers (35.88 %), and administrative staff (37.20 %). Finally, the respondents’ education levels included post-graduate (29.90%), undergraduate (48.17%), and others (21.92%). The detailed sample demographics are presented in Table 1.

## 5. Statistical Analysis

We used structural equation modeling (SEM) through SmartPLS 3.2 to assess the relationships in the research model [71]. We selected SmartPLS for our analysis because it follows the variance-based SEM approach, which is comparatively less sensitive to sample size than other applications that use covariance-based SEM approaches, such as AMOS (Analysis of Moment Structures) [72]. In this study, we tested the relationship between a toxic workplace environment, organizational support, employee well-being, and employee engagement. Therefore, before testing the hypothesized relationships, the reliability and validity of each construct were examined.

The construct reliability and validity values are presented in Table 2. The factor loading of each item was greater than the threshold value of 0.70. Similarly, Cronbach’s alpha, rho_A, and composite reliability measures for each of the constructs were higher than the recommended value of 0.7. Moreover, the average variance extracted for each construct was higher than the recommended value of 0.5 [73]. We also measured the reliability of all used constructs and quantified the convergent validity. The discriminant validity was tested. The square root of the average variance extracted for each construct should be higher than the shared variance among constructs. Hence, the scale fulfills the reliability and validity requirements.

Recently Henseler, Ringle [74] criticized the Fornell and Larcker [75] measurement standard and suggested that it is not reliable to measure discriminant validity. Henseler, Ringle [74] suggested another approach, based on the HTMT (Multitrait-Multimethod Matrix), to measure discriminant validity. It is a new technique to measure discriminant validity. In this study, we used the HTMT for the measurement of discriminant validity. The HTMT is defined as the mean value of the item correlations across constructs relative to the (geometric) mean of the average correlations for the items measuring the same construct. Henseler, Ringle [74] proposed a standard value of 0.90 for the HTMT. So, an HTMT value above 0.90 would suggest that discriminant validity is not present. Table 3 presents the HTMT value of each construct. The results indicat that the HTMT value of each construct is less than 0.90. So, the scale fulfills the discriminant validity requirements.

### Hypotheses Testing

**Figure 2:**
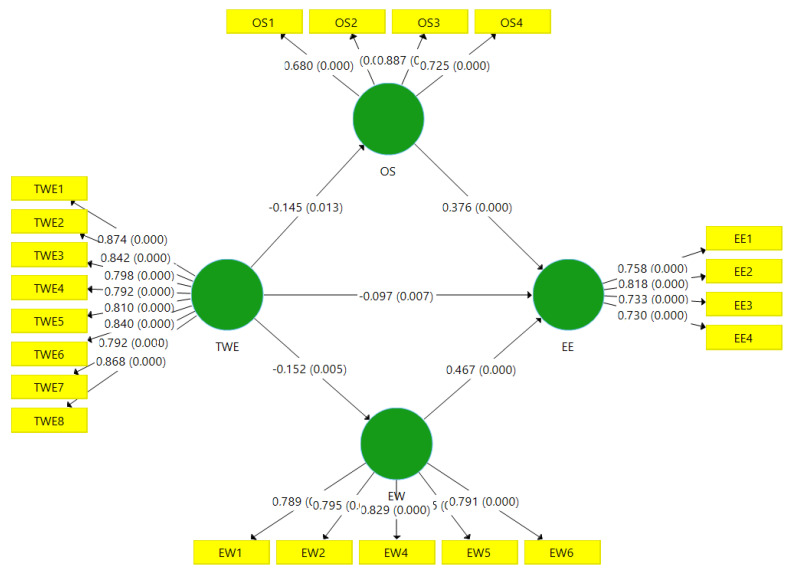
Path coefficients of the research model.

We used SmartPLS 3.2, with a bootstrapping technique, to calculate the path estimates and corresponding *t*-values, *p*-values, and confidence intervals [71]. The direct, indirect, and total effects of various relationships in the conceptual model, along with *t*-values, *p*-values, and confidence intervals, are presented in Table 4. The results indicate that a toxic workplace environment has a significant and negative relationship with employee engagement (β = −0.097, *p* < 0.05). So, hypothesis H1 of this study was accepted. Furthermore, a toxic workplace environment had a significant and negative relationship with organizational support (β = −0.145, *p* < 0.05). The results supported hypothesis H2a. Organizational support had a significant and positive relationship with employee engagement (β = 0.376, *p* < 0.05). Thus, hypothesis H2b was accepted. Organizational support mediated the relationship between a toxic workplace environment (TWE) and employee engagement (EE) (β = −0.062, *p* < 0.05). Hence, hypothesis H2c was accepted. Moreover, a toxic workplace environment was negatively related to employee well-being (β = −0.152, *p* < 0.05). Consequently, hypothesis H3a was also accepted. Employee well-being was positively related to employee engagement (β = 0.467, *p* < 0.05). Accordingly, hypothesis H3b of this study was also accepted. Lastly, employee well-being mediated the relationship between a TWE and EE (β = −0.061, *p* < 0.05), and the finding supported hypothesis H3c. Hence, the overall results of this study supported the hypotheses listed in Table 4. Moreover, the detail information of path coefficients of the research model is also present in Figure 2. 

## 6. Discussion

A toxic workplace environment and employee engagement have attracted the attention of many researchers. Previously, such kind of studies were conducted in advanced nations. This is the first study to be conducted in an emerging nation like China. Moreover, it is also the first study to be conducted amongst workers in small and medium-size enterprises of Guangdong Province, China.

First, the direct impacts of a toxic workplace environment on employee engagement were determined, and the findings of this research show that a toxic workplace environment has a negative influence on employee engagement, which support hypothesis H1. Prior studies also confirmed that a toxic workplace environment is negatively associated with employee engagement [76,77]. Similarly, a large-scale survey was conducted by Rasool, Maqbool [67] that was related to China’s banking sector. The results of their study confirmed that a toxic workplace environment has a negative connection with employee engagement, which reduces the individual worker’s productivity. The findings of this research are also supported by COR theory [55]. Furthermore, it is also noted that the workers’ health is affected by high job demands and work pressures; as a result, effects such as headaches, personality disorders, anxiety disorders, insomnia, burnout, and depression occur. So, it is suggested that some possible solutions could become the source of reduction in a toxic workplace environment for the workers in SMEs, which will ultimately increase employee engagement. Small and medium-size enterprises need to identify bad employees who are the root cause of the toxic workplace environment and then provide him/her with training on soft skills [68]. Second, SMEs should communicate with all functional heads, including supervisors, that the employees are the backbone of small and medium-size enterprises. So, they should be treated like an asset of the organization. These actions will reduce the toxic workplace environment and enhance employee engagement among the workers in small and medium-size enterprises in China.

Second, we inquired about the negative relationship between a toxic workplace environment and organizational support. The finding of this study confirms the negative relationship between a toxic workplace environment and organizational support, which supports hypothesis H2a. Wang, Zaman [32] suggested in their study that workplace violence reduces the support from organizations. The findings of this study are also supported by organizational support theory [78]. Moreover, in this study, we tested the positive relationship between organizational support and employee engagement. The finding of this investigation endorses that there is a positive relationship between organizational support and employee engagement, which supports hypothesis H2b. Tremblay, Gaudet [79] examined 115 business units of an international retailer, and the findings of their research showed that organizational support is positively linked to employee engagement. These findings are also supported by organizational support theory [53]. We also investigated the intervening role of organizational support in the relationship between at oxic workplace environment and employee engagement. The finding of this research confirms that organizational support mediates the relationship between a toxic workplace environment and employee engagement, which support hypothesis H2c. Past studies also support our results [80,81]. Organizational support theory also supports our results [82]. Organizational support theory suggests that people trade their time and effort at work for valued outcomes. So, if Chinese small and medium-size enterprises provide market-based compensation and benefits, then workers will be satisfied and engage with the vision of the organizations. Similarly, Chen, Hao [83] demonstrated that organizational support is the degree to which workers think that their organization values their contribution and cares about their well-being.

Third, we investigated the negative relationship between a toxic workplace environment and employee well-being. The results of this research confirm the negative relationship between a toxic workplace environment and employee well-being, which supports hypothesis H3a. Samma, Zhao [2] examined 254 workers employed at small and medium-size enterprise (SMEs) located in Pakistan, and the outcomes of their study indicated that a toxic workplace environment negatively influences employee well-being. Specifically, in this relationship, COR theory confirms that most of SMEs are facing workplace violence, which brings about a negative attitude among the workers, which negatively affects the balanced and emotional well-being connection with the vision and mission of the organization [55]. Moreover, in this study, we tested the positive relationship between employee well-being and employee engagement. The findings of this paper confirm that there is a positive relationship between organizational support and employee engagement, which supports hypothesis H3b. The evidence of Shuck and Reio Jr. [84] suggested that a worker’s well-being goes hand in hand with worker engagement. When employers provide health and financial support, employees give their full attention at work. The findings of this study are also supported by organizational support theory [78]. Furthermore, we used employee well-being as a second mediating variable in the relationship between at toxic workplace environment and employee engagement. The outcomes of this research confirm that employee well-being positively and significantly mediates in the relationship between a toxic workplace environment and employee engagement, which supports hypothesis H3c. Zhou, Rasool [55] examined 336 workers employed at small and medium-size enterprises, and the results of their study confirmed that employee well-being reduces workplace harassment, mobbing, and sabotage. As a result, it improves innovative work behavior, which increases employee engagement. Similarly, previous studies also support our results [85,86,87]. This relationship is supported by organizational support theory [88]. So, the above discussion proves that organizational support and employee well-being reduce a toxic workplace environment and increase the level of employee engagement. Additionally, it is also proven that employee well-being is more significant than organizational support.

## 7. Conclusions

In this study, first, we checked the direct relationship between a toxic workplace environment and employee engagement. So, the results of this study confirm that there is a negative relationship between a toxic workplace environment and employee engagement. Similarly, we tested the indirect relationship between a toxic workplace environment and employee engagement using two mediating variables, i.e., organizational support and employee well-being. The results also confirm that employee well-being and organizational support significantly mediate the relationship between a toxic workplace environment and employee engagement. The negative relationships are supported by COR theory, and the positive relationships are supported by organizational support theory.

The conclusions of this paper are as follows: First, the direct relationship between a toxic workplace environment and employee engagement confirms that if employees are working in a toxic environment, they will spread negative feelings among other co-workers. The feelings that come with a toxic workplace environment, i.e., harassment, bullying, and ostracism, can be detrimental and lead to unnecessary stress, burnout, depression, and anxiety among the workers. Second, employee well-being will affect employee behaviors that enhance employee engagement with the work as well as with the organization. Employee engagement creates harmony in the organization. Moreover, employee well-being increases the workers’ work performance. Furthermore, when an organization works for the well-being of the workers, it reduces the toxic workplace environment and brings sustainability to organizational performance. Third, organizational support also increases employee engagement with the work as well as with the organization. Finally, this study also noted that workers want to contribute to their organizations. It is usually a matter of agreeing on a vision, finding the right fit for their particular contributions, and being open to feedback. So, organization custodians listen first and then work with management teams to develop goals and expectations that can be celebrated when they are achieved. It is a four-step process: engage, enlighten, empower, and evolve together. Moreover, employee engagement aligns employees’ personal goals with the vision of small and medium-size enterprises, which increases the productivity of small and medium-size enterprises’ employees and sustainable organizational performance. An engaged employee is well balanced and emotionally connected with the vision and mission of the organization, which portrays and governs the involvement of the employee in the organizational objectives.

## 8. Limitations and Future Research

The study had certain limitations. First, the sample size was small. A larger sample size will provide a more diversified sample that ought to be used to test the proposed model in future research for a further extension of the validity of the end results. Second, this study only investigated small and medium-size enterprises located in China. In the future, such kind of research can be conducted in another country or can be conducted in another industry such as healthcare, automobile, construction, and information technology. Third, in this study, we did not consider the gender effect in the relationship between a toxic workplace environment and employee enjoyment. So, in the future, gender effect as a mediating variable or moderator can be tested. Lastly, in this research, we used COR theory for negative relationships and organizational support theory for positive relationships. In the future, researchers can use a resource-based view (RBV) or knowledge-based view (KBV) in the present framework. As a result, future research could be exploratory toward the relationships amongst these factors and workplace violence and sustainable organizational performance using job anxiety or job burnout as a mediating variable.

## Figures and Tables

**Table 1:** Respondents’ summary.

Characteristics	Category	Frequency (*n*)	Percentage (%)
Gender	Male	164	54.44
	Female	137	45.51
Working experience	5–10 years	119	39.53
	10–15 years	102	33.88
	Above 15 years	80	26.57
Positions	Senior manager	81	26.91
	Middle manager	108	35.88
	Administrative staff	112	37.20
Education	Post-graduate	90	29.90
	Undergraduate	145	48.17
	Others	66	21.92

**Table 2:** Reliability and validity of the construct.

Constructs	Loading	Alpha	rho_A	CR	AVE
**Toxic Workplace Environment**		0.935	0.94	0.946	0.685
TWE1	0.874				
TWE2	0.842				
TWE3	0.798				
TWE4	0.792				
TWE5	0.810				
TWE6	0.840				
TWE7	0.792				
TWE8	0.868				
**Organizational Support**		0.784	0.795	0.862	0.612
OS1	0.680				
OS2	0.820				
OS3	0.887				
OS4	0.725				
**Employee Well-Being**		0.843	0.846	0.889	0.616
EW1	0.789				
EW2	0.795				
EW3	0.829				
EW4	0.716				
EW5	0.791				
**Employee Engagement**		0.759	0.776	0.846	0.578
EE1	0.758				
EE2	0.818				
EE3	0.733				
EE4	0.730				

**Note:** TWE, toxic workplace environment; EE, employee engagement; OS, organizational support; WB, employee well-being; CR, composite reliability; rho_A, Dijkstra–Henseler’s rho (ρA); AVE, average variance extracted.

**Table 3:** Discriminant validity.

Constructs	EE	EW	OS
Employee engagement			
Employee well-being	0.786		
Organizational support	0.759	0.533	
Toxic workplace environment	0.244	0.157	0.166

Notes: Significant level *p* < 0.05. EE, employee engagement; EW, employee well-being; OS, organizational support.

**Table 4:** Direct and indirect paths.

Direct Paths	Coefficients	Mean	SD	*t*-Values	*p*-Values	Results
TWE → EE	−0.097	−0.098	0.037	2.590	0.010	Significant
TWE → OS	−0.145	−0.155	0.054	2.812	0.005	Significant
OS → EE	0.376	0.378	0.044	8.554	0.000	Significant
TWE → EW	−0.152	−0.152	0.059	2.465	0.014	Significant
EW → EE	0.467	0.466	0.05	9.378	0.000	Significant
**Indirect Paths**						
TWE →EW → EE	−0.062	−0.068	0.024	2.601	0.009	Significant
TWE → OS → EE	−0.061	−0.067	0.023	2.606	0.009	Significant

Note: Significant level *p* < 0.05. SD, standard deviation; TWE, toxic workplace environment; EE, employee engagement; OS, organizational support; WB, employee well-being.

## Data Availability

The data will be available on request.

## Appendix A

	**Research Instrument**
	**Toxic Workplace Environment**
**1.**	My supervisor/co-worker/subordinate often appreciates my physical appearance.
**2.**	My supervisor/co-worker/subordinate spoke rudely to me in public.
**3.**	My supervisor/co-worker/subordinate often tries to be frank with me and shares dirty jokes with me.
**4.**	My supervisor/co-worker/subordinate assigns me work that is not of my competence level.
**5.**	My supervisor/co-worker/subordinate often tries to talk about my personal and sexual life.
**6.**	My supervisor/co-worker/subordinate tries to maintain distance from me at work.
**7.**	My supervisor/co-worker/subordinate does not answer my greeting.
	**Organizational Support**
**8.**	The organization attaches great importance to my work goals and values.
**9.**	The organization always helps me whenever I am facing a bad time.
**10.**	The organization is flexible with my working hours, if needed, whenever I guarantee to complete my tasks on time.
**11.**	The organization provides me enough time to deal with my family matters.
	**Employee Well-Being**
**12.**	I generally feel positive toward work at my organization.
**13.**	My supervisor and co-worker check in regularly enough with how I am doing.
**14.**	When I am stressed, I feel I have the support available for help.
**15.**	Our organizational culture encourages a balance between work and family life.
**16.**	Our organization provides aid in stress management.
	**Employee Engagement**
**17.**	I really throw myself into my job and organization engagement.
**18.**	I fulfil all responsibilities required by my job.
**19.**	I willingly give my time to help others who have work-related problems.
**20.**	I always complete the duties specified in my job description.
